# Boosting Advanced Nasopharyngeal Carcinoma Stage Prediction Using a Two-Stage Classification Framework Based on Deep Learning

**DOI:** 10.1007/s44196-021-00026-9

**Published:** 2021-10-19

**Authors:** Jin Huang, Ruhan He, Jia Chen, Song Li, Yuqin Deng, Xinglong Wu

**Affiliations:** 1 School of Computer Science and Artificial Intelligence, Wuhan Textile University, Wuhan, 430200 Hubei China; 2 Department of Otolaryngology-Head and Neck Surgery, Renmin Hospital of Wuhan University, Wuhan, 430060 Hubei China; 3 School of Computer Science and Engineering, Wuhan Institute of Technology, Wuhan, 430205 Hubei China

**Keywords:** Two-stage classification, Deep learning, Interpretability, Advanced nasopharyngeal carcinoma, MRI, stage prediction

## Abstract

**Abstract:**

Nasopharyngeal carcinoma (NPC) is a popular malignant tumor of the head and neck which is endemic in the world, more than 75% of the NPC patients suffer from locoregionally advanced nasopharyngeal carcinoma (LA-NPC). The survival quality of these patients depends on the reliable prediction of NPC stages III and IVa. In this paper, we propose a two-stage framework to produce the classification probabilities for predicting NPC stages III and IVa. The preprocessing of MR images enhance the quality of images for further analysis. In stage one transfer learning is used to improve the classification effectiveness and the efficiency of CNN models training with limited images. Then in stage two the output of these models are aggregates using soft voting to boost the final prediction. The experimental results show the preprocessing is quite effective, the performance of transfer learning models perform better than the basic CNN model, and our ensemble model outperforms the single model as well as traditional methods, including the TNM staging system and the Radiomics method. Finally, the prediction accuracy boosted by the framework is, respectively, **0.81**, indicating that our method achieves the SOTA effectiveness for LA-NPC stage prediction. In addition, the heatmaps generated with Class Activation Map technique illustrate the interpretability of the CNN models, and show their capability of assisting clinicians in medical diagnosis and follow-up treatment by producing discriminative regions related to NPC in the MR images.

**Graphic Abstract:**

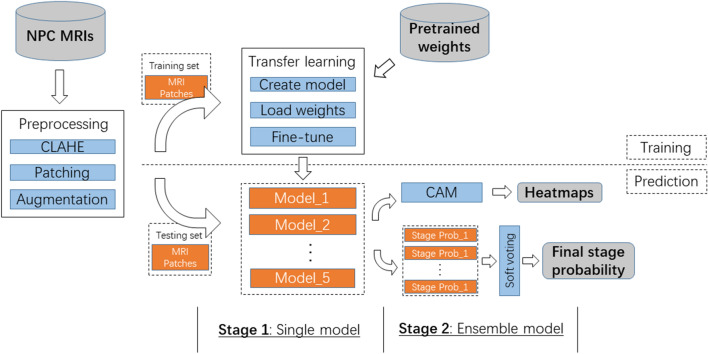

## Introduction

Nasopharyngeal carcinoma (NPC) is a popular malignant tumor in the world. Stage describes the growth and spread of the NPC. There is a significant difference in the survival rate of NPC patients in the early stages (I, II) and late stages (III, IV). Different treatment options need to be selected for the patients in different stages. Radiotherapy is the key treatment for early-stage NPC [1], while the combination of radiotherapy and chemotherapy is the main treatment strategy for advanced NPC. Over 75% of patients suffer from locoregionally advanced Nasopharyngeal Carcinoma (LA-NPC) in stage III or IVa at the first diagnosis, where concurrent chemoradiotherapy (CCRT) and induction chemotherapy (IC) are suggested. However, aiming at improving the survival quality of the patients with advanced NPC, aggressive treatment options are not recommended for any patients in stage III/IVa [2]. The study declares that CCRT is sufficient for low-risk patients of LA-NPC (stage III), while additionally IC is required for high-risk patients (stage IVa). Therefore, an accurate treatment plan depends on effective LA-NPC stage prediction.

The LA-NPC stage prediction is a critical function of computer-assisted diagnosis (CAD) systems for NPC patients. CAD system is used to analyze different medical data to assist clinicians in diagnosis. It can reduce the burden on clinicians and accelerate the diagnosis process, but not replace the role of professional doctors. Nowadays, various medical images are widely analyzed by different machine learning methods including computer vision methods.

Techniques for NPC stage prediction have been developed over decades. The most popular method of NPC stage prediction used in hospitals is the tumor-node-metastasis (TNM) staging system. The system requires experienced clinicians to collect and analyze various data to determine the stage of each patient very carefully. Radiomics is another technique that achieves automatic NPC stage prediction using medical images and clinical data. This technique typically needs to perform tumor segmentation on the original image as preprocessing, extracts features from its output, and achieves classification for stage prediction. Recently, deep learning techniques have been used for cancer classification. The data set of medical images is used to train the classification model, and then the medical images of patients are fed into the model to generate classification probabilities for different cancer stages. The LA-NPC are classified into two different stages (III, IVa). The study [2] achieves the LA-NPC stage prediction using the deep learning model, while ensemble learning is used to improve the prediction performance. Besides, CAM [4] takes advantage of the class-specific gradient of CNN to produce discriminative regions related to specific cancers in MR images, and these highlighted regions can assist clinicians in diagnosis and follow-up treatment.

Most MR images of NPC patients available in the hospital are 2D axial slices in the positions of neck and nose, and the slices are not that dense (intervals between 2 and 7mm) to preserve enough 3D features across slices. In MR images of NPC patients, organs and tissues around the tumors are of great diagnosis significance for predicting stages of cancer by radiologists [3]. However, segmentation removes the surrounding tissues that are indispensable for stage prediction. Besides, there are usually not enough MR images to train a deep learning model from scratch. In addition, deep learning is still considered a black-box technique that should be interpretable for critical applications, such as cancer diagnosis and prognostic prediction.

In this paper, we believe that 2D-CNN is a reasonable classification model to analyze MR images for LA-NPC stage prediction. Due to the lack of images, transfer learning is to improve the model training efficiency and prediction effectiveness. The intuition is that various tumors can be classified or detected through their shapes and texture features, that are similar to the features of nature images in data sets, such as ImageNet. The pretrained models are well trained to capture low level features so that they are to achieve stage prediction using minor fine-tuning with limited images. Furthermore, ensemble learning is to boosting the classification performance with different weak classifiers. Classification probabilities produced by different 2D-CNN models can be ensembled using a voting method to boost the final NPC stage prediction.

### Contributions

The main contributions of this paper are listed as below:

1. A novel image preprocessing method is proposed to enhance the quality of MR images for NPC stage prediction. According to the quality, complexity and limited size of MR images of NPC patients, we use several images operations to reduce the negative effect of data set for model training.
2. Transfer learning is used to improve the effectiveness of prediction, as well as the efficiency of model training. The pretrained models are well trained to capture various low level features, which is of significant benefit to the training of CNN models for stage prediction.
3. We build a two-stage classification framework to improve NPC stage prediction. All prediction results of different advanced CNN models are then ensembled using soft voting to boost the final classification performance. As far as we know, there is no such research using ensemble framework based on CNN models for NPC stage prediction.

The rest of this paper is organized as follows. In Sect. 2, we give the recent studies related to NPC stage prediction. Sect. 3 describes our stage prediction method. The experimental results and comparisons are discussed in Sect. 4. Conclusions and future work are given in Sect. 5. We will make code and part data publicly available from our projects webpage, https://github.com/hj0320/NPC-stage-prediction.

## Related Work

### NPC Stage Prediction

The stage prediction is a critical diagnostic method for selecting follow-up treatment options. TNM staging system [5] is one of the most popular methods to predict the NPC stages. Experienced clinicians predict the stages for patients using various types of data including clinic information and features of medical images.

Radiomics [6] is another popular technique to predict the stages in oncology. In recent years, more and more machine learning-based studies on NPC diagnosis have been reported [7–10]. A data analysis framework with prognostic factors [7] including radiomics features of multi-parametric MR images and clinical data is built to improve prognostic ability in LA-NPC. The study shows that Radiomics features from medical images could be effective prognostic factors for NPC diagnosis. The research [8] explores the quantitative features of multi-modalities MR images and evaluates the feasibility of radiomics in classifying NPC into survival subgroups. The clustering technique is to generate a baseline model as a classification reference, while the support vector machine (SVM) model is trained as a classifier to predict survival subgroups. The study [9] establishes a radiomics nomogram to predict induction chemotherapy (IC) response and survival. The radiomics nomogram is built by fusing the clinical data and Radiomics features of multi-parametric MR images with a SVM. Recently, the research [10] predicts the NPC prognostic value with radiomics features generated by deep learning technique. Taking advantage of the radiomics features and clinical prognostic information, a Radiomic nomogram is constructed for pretreatment prognostic prediction. However, the existing methods for NPC stage prediction require the step of tumor segmentation as the preprocessing step, which make the diagnosis more time-consuming and error-prone. Moreover, the segmentation removes the valuable information from medical images. It is doubtful that such related tissues and organs around the tumors are removed insouciantly.

Deep learning based classification methods are also used to predict cancer stages [2, 11, 12]. To achieves the LA-NPC stage prediction with MR image stacks, the study [2] has trained a classifier based on 3D-CNN model using a large data set of MRI stacks. However, in most hospital there is a few 2D MR image taken for each NPC patient, unfortunately there is few research on using 2D-CNN models to analyze the 2D images for stage prediction. Besides, due to the lack of large labeled data set, a weakly supervised method [11] is developed to predict the T stage with few labels. Even though the staging of penile cancer is achieved through the TNM staging system by clinicians, deep-learning using medical images is an automatic end-to-end solution for stage prediction [12].

### Ensemble Learning

Ensemble learning methods including boosting [13], stacking [14] and voting [15] are effective machine learning techniques to improve the performance of medical image classification.

2D-DWT [16] is utilized to extract features in medical images for detecting abnormality in brain. An automatic classifier is proposed with AdaBoost and random forests. The study [17] extracts texture-based features from MR images with GLCM technique and achieves brain tumor classification with Adaboost, while the Adaboost classifier achieves the accuracy of 89.90%. Boosting-based technique [18] is able to predict or classify from non-image clinical data, including 9 different types of disease such as breast cancer, mental health survey, cleveland heart et al.

In recently years, [19, 20] review advances in deep multimodal learning, as well as the application of stacking technique in this research field. Deep multimodal learning methods are reviewed and deep fusion methods for multimodal representations are discussed. Liu [21] presents a multimodal classification algorithm based on the stacked CNNs to predict Alzheimers Disease (AD) and mild cognitive impairment (MCI) from normal control (NC) with MR and PET images. The study [22] proposes a multi-modality VGG-based model and stacking technique using the hippocampal area of no segmentation as the input. The work [23] investigates the suitability of a modified residual neural network (ResNet) and stacked model for studying brain MR and PET images for predicting progression from MCI to AD. The study [24] achieves the MediaEval Medico task through an ensemble learning method with multiple classifiers, including traditional machine learning methods and CNN-based classifiers.

### Deep Learning

Recently, deep learning techniques have been widely used to classify various medical images, such as X-ray, CT and MRI. Nowaday, various convolutional neural network models such as VGG [25], ResNet [26], and InceptionResnetV2 [27] are widely used to improve performance of different computer vision tasks, including image classification [28], object detection [29], Biomedical image segmentation [30] and image enhancement [31] in low-light environment. Meanwhile, more and more researches attempt to use CNN models to improve the accuracy of diagnosis with medical images [32], help reconstruction of CT and MR images [33], as well as GTV (Gross Tumor Volume) Delineation for radiologists [34].

Besides, due to the lack of data annotations in the target domain, transfer learning is to optimized machine learning models by taking advantage of data set in the source domain [35]. Transfer learning is widely used for medical image analysis [36] due to the unavailability of such large labeled data set. Recent researches on COVID-19 prediction [37, 38] analyze the lung images using transfer learning based on deep learning.

Finally, a series of class activation map (CAM) techniques [39–42] has been developed to highlight the region of high response for the specific class to explain the results of a classifier. In medical image analysis, this technique helps clinicians quickly locate the region of interest, as well as interpret the effectiveness of the methods [43, 44].

## Materials and Methodology

### Data Set

This research was approved by the Ethics Committee of the Renmin Hospital of Wuhan University, and the informed consent from patients has been exempted. The data set for this study is collected from the patients with NPC diagnosed for treatment in Renmin Hospital of Wuhan University from 2012 to 2018. According to the statistics for these patients, about 75% of them suffer from LA-NPC, and they are classified into a low-risk group and a high-risk group labeled with different stages (III, IVa). Finally, More than 200 patients including males and females are selected. Their medical records include gender, age, clinical data, MR images, TNM staging, and survival data. The stage of each patient has been checked by at least two experienced clinicians according to his/her medical records.

**Fig. 1:**
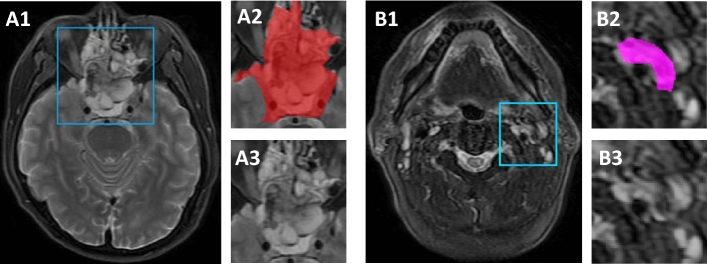
MRI examples of LA-NPC including tumors and lymph nodes. **A1**  Full-field MRI of tumor, **A2**  tumor with its mask, **A3**  MRI patch of tumor; **B1**  full-field MRI of lymph node, **B2**  lymph node with its mask, *B3*  MRI patch of lymph node

More than 20,000 patches of tumors and lymph nodes are cropped out of the full-field MR images by clinicians, and the examples are shown in Fig. 1. Since clinicians need the images on the nasopharyngeal and neck positions, about only 20 slices of each patient are kept for the medical diagnosis. The Axial MRIs of enhanced T1 and WATER T2 are selected for further processing. Rectangular regions of interest (ROI) in each MRI are cropped with the appropriate resolutions from $50 \times 50$ to $300 \times 300$. In addition to tumors and lymph nodes, the area around them is also preserved, so that the tissue surrounding them is also kept in the patches for stage prediction. The resolutions of the patches can be different, since the sizes of tumors are different from each other.

The whole data set is split into the training set and testing set with the ratio of 4:1, as listed in Table 1. Train_III is the training set for the LA-NPC of stage III, while Test_III is the testing set for stage III. Train_IVa is the training set for stage IVa, while Test_IVa is the testing set for stage IVa. The patches of both tumors and lymph nodes are collected into the data set. Finally, the patch numbers of all subsets are given in the last column, and the sizes of data sets for the two stages are similar. If the researchers need the whole data set, please contact the Renmin Hospital of Wuhan University through the email *bamboo0723@126.com* for more information.

**Table 1:** LA-NPC MR image data set

Data set	Tumors	Lymph nodes	Total
Train_III	5969	2185	8154
Train_IVa	6820	2217	9037
Test_III	1492	546	2038
Test_IVa	1705	554	2259

### Framework

**Fig. 2:**
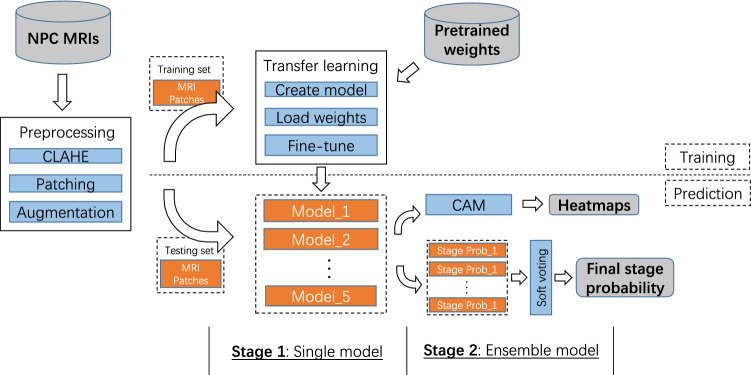
Pipeline of our two-stage classification framework. All MR images of NPC patients are reprocessed to produces MRI patches for the following model training and prediction. During training, CNN models are fine-tuned through transfer learning. During prediction, in the first stage MRI patches are fed into CNN models to output stage probabilities, while in the second stage these probabilities are ensembled to produce final stage probability for each MRI patch

We present our two-stage classification framework based on the advanced CNN models for the stages prediction, as shown in Fig. 2. Before the training of the models, the MR images are preprocessed in three steps. First, the original MR images are enhanced with Contrast Limited Adaptive Histogram Equalization (CLAHE) to optimized their contrast. Then the patches of tumors and lymph nodes are cropped manually by experienced clinicians from these images. Finally, these patches are augmented to increase the size and diversity of the existing data set, then resized as inputs of following classifiers.

This unified framework mainly consists of a single model stage and a ensemble model stage. During model training, we load the pretrained weight of each CNN model and fine-tune the model with preprocessed MRI patches as input in the first stage, which is a typical transfer learning method of training the medical machine learning models taking advantage of general models. During the process of prediction, in the first stage each MR image patch is fed into different CNN models separately to output stage probabilities which is the classification probabilities of being two different NPC stages. Then, in the second stage, these probabilities are ensembled with a voting method to produce the final stage probability for each MRI patch. In this stage, ensemble learning are crucial machine learning techniques to improve the performance of training and prediction. In addition, the heatmaps of MR images are generated with the CAM technique by taking advantage of specific layer of CNN models.

### Preprocessing

**Fig. 3:**
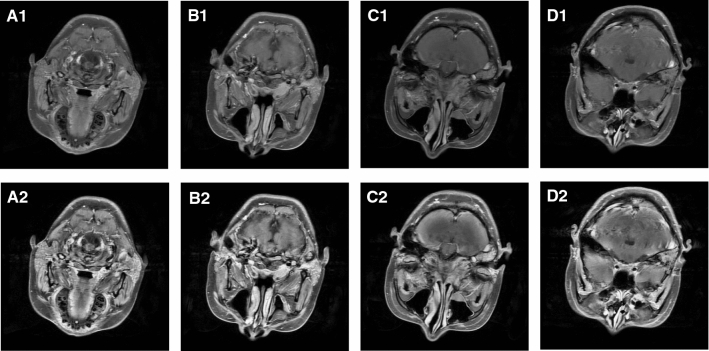
MRI examples before and after preprocessing. The images in the first row are original ones, while the images in the second row are processed using CLAHE

Brain MR images show the internal structure of the brain with contrast variation in gray scale. Since the image acquisition is performed with different equipment or under different conditions, the contrast of the MR images may not be ideal, which will have a negative impact on the prediction result. Image normalization enhances image quality to improve the robustness of the CAD system. More precisely, normalization can be used to reduce the potential noise in the data set to avoid overfitting for building a robust classification method. CLAHE is one of the histogram equalization methods especially designed for MR image enhancement. With the help of CLAHE, the MR images are enhanced significantly for further analysis. Some MRI examples of NPC are shown in Fig. 3. The images in the first row are original ones, and in the second row have been preprocessed with CLAHE, which enhances the contrast and the texture of various organs and tissues in MR images.

Due to the complexity of MR images for NPC, it is difficult to predict stages using the original full-field images. Unlike MR images for a brain tumor or knee disease, there are much more organs of complex structures in the positions of the human nose and neck. One solution is to intercept the ROIs in the images to improve the classification performance, so the patches of tumors and lymph nodes are cropped from full-field images by 2 experienced clinicians and checked by another one. In addition, the tissues surrounding the tumors and lymph nodes are useful for NPC stage prediction. To preserve the related tissues, the resolution of each patch is larger than that of the tumor or lymph node. Finally, there may be several tumors or lymph nodes in one MR image, so the total number of patches is several times the MR images.

Data augmentation is one of the best strategies to avoid overfitting. When the size of the data set is small, it is required to increase its size and variety. There is the limited size of medical data, such as MR images, since the confirmed cases are rare and the data acquisition is difficult. Data augmentation generates more images from the original data set with various image operations including rotation, shifting, shearing, zooming, and flipping. These operations are reasonable, since the positions of patients may be a little different during acquisition, also, tumors and lymph nodes are different by size and shape. A data set of large size and high variety is critical for improving model generalization and building a robust classification model for medical applications.

### Single Model

Deep CNN model allows to learn low-level features, such as edges, corners, and intensity from images using bottom layers, these features can be shared across different tasks. Taking advantage of the bottom layers, knowledge transfer is enabled among tasks. The high-level features such as shapes and objects are extracted by the following pooling layers and convolutional layers. With the help of top layers including fully connected layers, the deep learning model can finally produce classification result. The performance of models highly depends on the data set available. The advanced CNN models (VGG16, InceptionV3, ResNet50, InceptionResNetV2, and DenseNet121) are well trained with large data sets,such as ImageNet. We can freeze the convolutional layers and fine-tune the top layers to classify new classes using data of small size in a few epochs, since the low-level features of new classes are similar to that of the existing class in ImageNet, and the bottom layers are able to extract the low-level features efficiently without any training. Even though the medical image data set is of small size, the low-level features are similar so that transfer learning can achieve ideal classification performance using pretrained models.

**Fig. 4:**
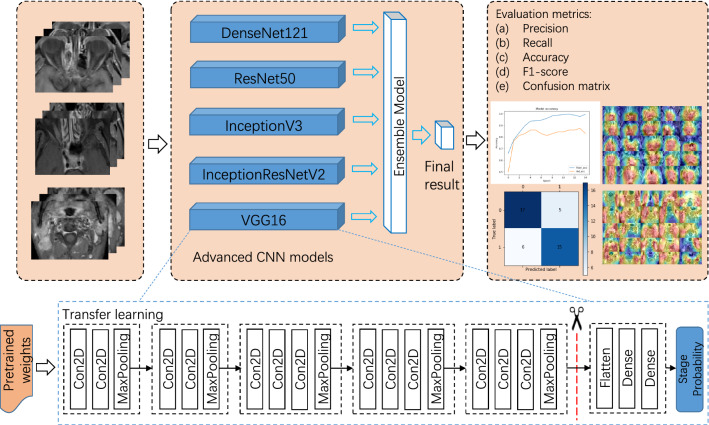
Model for transfer learning and ensemble learning

We take the fine-tuning of the VGG16 model as an example to illustrate the transfer learning process, as shown in Fig. 4. We create a VGG16 model without top layers and load its pretrained weights trained with ImageNet. Then we add the top layers for binary classification, since our task is to predict two classes. We freeze the model except for the top layers and train the model using our MRI data set. After several epochs the training is stopped and the model weights are saved for model testing and prediction. The transfer learning processes of the other 4 advanced models are similar to VGG16 and achieved independently.

### Ensemble Model

Ensemble learning is used, while different classification models exist and produces a better a result than single model in most cases. In the ensemble model stage of our framework, voting is used to ensemble the output of five advanced CNN models to improve the final classification performance, as shown in Fig. 4. During prediction, each patch is classified with the advanced CNN models, then their outputs are ensembled to get the final classification result for the stage prediction.

Soft voting is used to build a classifier with a majority vote of *m* basic classifiers, while $m=5$ in this study, since five advanced CNN models are used. The number of class types $k=2$, since the task is a binary classification. We choose soft voting as our voting method. For soft voting, the class probability of MR images is given by Eq. (1), where $p_i^k$ is the class probability vector for basic classifier $i (1 \le i \le m)$, $p^k$ is the class probability vector of all *k* classes. Finally, the class assignment with soft voting classifier is given by Eq. (2), where *argmax* returns an index $c (1 \le c \le k)$ of the maximum value in given probability vector $p^k$.

1 $$\begin{aligned} p^{k}= & {} \frac{1}{m} \sum _{i=1}^{m} p_{i}^{k}, \end{aligned}$$

2 $$\begin{aligned} c= & {} \arg \max p^{k}. \end{aligned}$$

## Evaluations and Comparisons

In this section, we first introduce the settings of our experiments. Then we give experimental analysis to explain the advantage of the advanced CNN models comparing with the basic CNN model. We also compare our ensemble model with the advanced CNN models. Finally, the heatmaps of MR images are presented and discussed.

### Experimental Setup

All the experiments are achieved using NVIDIA GPU and Ubuntu, and the configures of the experimental platform are listed in Table 2. Keras is used to achieve training and testing with five pre-trained models downloaded from keras official website. The resolutions of the patches are different, so we resize them to the fixed resolutions. Before fine-tuning, the model weights are loaded, $binary\_crossentropy$ is chosen as loss function, the optimizer is set to *Adam*, and the initial learning rates of these models are set to 0.0001. The learning rate is updated by a scheduler. During the fine-tuning, *LearningRateScheduler* module of Keras is used to update the learning rate gradually with the increase of epoch number. The batch size for training is 32.

**Table 2:** Experimental platform configurations

Items	Configurations
CPU	Xeon E5 2620v3
GPU	Nvidia P5000
Memory	128G
Operating system	Ubuntu 16.04.5 LTS
CUDA	CUDA 9.0
Language	Python 3.6
Library	Keras 2.2, tensorflow-gpu 1.13

For the performance evaluation of the classification models, we use various metrics including accuracy (ACC), precision (PRE), sensitivity (SEN) and F1-score (F1). We calculate the metrics for both classes, and in this section we give all results of LA-NPC stages III and IVa. Sklearn modules are used to calculate the valuation metrics for both classes, and the formulas for calculating the metrics are listed below:

3 $$\begin{aligned}&\mathrm{ACC}=\frac{\mathrm{TP} + \mathrm{TN}}{\mathrm{TP} + \mathrm{TN} + \mathrm{FP} + \mathrm{FN}}, \end{aligned}$$

4 $$\begin{aligned}&\mathrm{PRE}=\frac{\mathrm{TP}}{\mathrm{TP} + \mathrm{FP}}, \end{aligned}$$

5 $$\begin{aligned}&\mathrm{SEN}=\frac{\mathrm{TP}}{\mathrm{TP} + \mathrm{FN}}, \end{aligned}$$

6 $$\begin{aligned}&\mathrm{F1}=\frac{2}{1/\mathrm{PRE} + 1/\mathrm{SEN}}. \end{aligned}$$

### Evaluation Results

#### Effect of MR Image Preprocessing

We compare the NPC stage IVa prediction results of different CNN models with and without MR image preprocessing, all scores are listed in Table 3. With the preprocessing, the VGG16 model achieves a higher precision of 0.78, a higher sensitivity of 0.75,a higher F1-score of 0.76, and a higher accuracy of 0.75. With the preprocessing, the InceptionV3 model achieves a higher precision of 0.75, a higher sensitivity of 0.75,a higher F1-score of 0.75, and a higher accuracy of 0.74. With the preprocessing, the ResNet50 model achieves a higher precision of 0.77, a higher sensitivity of 0.73,a higher F1-score of 0.75, and a higher accuracy of 0.73. With the preprocessing, the InceptionResNetV2 model achieves a higher precision of 0.82, a higher sensitivity of 0.76,a higher F1-score of 0.79, and a higher accuracy of 0.78. With the preprocessing, the DenseNet121 model achieves a higher precision of 0.76, a higher sensitivity of 0.81,a higher F1-score of 0.79, and a higher accuracy of 0.77. According to the comparison results, with the preprocessing step all models achieve better scores. Therefore, we strongly suggest the MR image preprocessing before further image analysis.

**Table 3:** Different models with or without MR image preprocessing, and prediction results for NPC stage IVa prediction

Models	Preprocess	Precision	Sensitivity	F1-score	Accuracy
VGG16	–	0.72	0.74	0.72	0.73
	$\checkmark$	**0.78**	**0.75**	**0.76**	**0.75**
InceptionV3	–	0.70	0.73	0.72	0.73
	$\checkmark$	**0.75**	**0.75**	**0.75**	**0.74**
ResNet50	–	0.72	0.70	0.71	0.69
	$\checkmark$	**0.77**	**0.73**	**0.75**	**0.73**
InceptionResNetV2	–	0.77	0.74	0.72	0.74
	$\checkmark$	**0.82**	**0.76**	**0.79**	**0.78**
DenseNet121	–	0.74	0.73	0.75	0.71
	$\checkmark$	**0.76**	**0.81**	**0.79**	**0.77**

Bold values indicate the better scores with and without preprocessing for each model

#### Effect of Transfer Learning

We give the results of basic CNN model and five advanced CNN models, then tell the difference between basic CNN model and the advanced CNN models. Since there is few research on stage prediction using 2D-CNN model, we build a basic CNN model as baseline for comparison. The basic CNN model has 3 convolutional layers, each layer is followed by a max-pooling layer, then 2 densely connected layers are added as top layers to produce classification result. All the testing results of basic CNN model and advanced CNN models are listed in Table 4.

**Table 4:** Different models for prediction NPC stages III/IVa, and all prediction results

NPC stages	Methods	Precision	Sensitivity	F1-score	Accuracy
Stage III	Basic CNN	0.68	0.71	0.69	0.69
	VGG16	0.73	0.76	0.75	0.75
	InceptionV3	0.72	0.72	0.72	0.74
	ResNet50	0.72	0.76	0.74	0.74
	InceptionResNetV2	0.75	0.81	0.78	0.78
	DenseNet121	0.77	0.72	0.75	0.77
	Ours	**0.79**	**0.83**	**0.81**	**0.81**
Stage IVa	Basic CNN	0.73	0.71	0.72	0.71
	VGG16	0.78	0.75	0.76	0.75
	InceptionV3	0.75	0.75	0.75	0.74
	ResNet50	0.77	0.73	0.75	0.73
	InceptionResNetV2	0.82	0.76	0.79	0.78
	DenseNet121	0.76	**0.81**	0.79	0.77
	Ours	**0.84**	0.80	**0.82**	**0.81**

Bold values indicate the best scores using different methods for stage III and IVa

Precision is a fundamental metric to evaluate the performance of classification. It tells the fraction of instances of a class among all instances predicted as the same class. A perfect classifier has precision equal to 1. For predicting stage III of LA-NPC, the precision of basic CNN model is only 0.68, the precisions of 5 advanced CNN models are all higher than 0.72. For predicting stage IVa, the precision of basic CNN model is only 0.73, the precisions of the advanced CNN models are all higher than 0.75. The result show that the precisions of the outlined advanced CNN models in this study are better than the basic CNN model.

Sensitivity(Recall) is another fundamental metric to evaluate the performance of classification. It tells the fraction of instances correctly classified among all instances of that class. A perfect classifier has a sensitivity equal to 1. For predicting stage III, the sensitivity of basic CNN model is only 0.71, the sensitivities of the advanced CNN models are all higher than 0.72. For predicting stage IVa, the sensitivity of basic CNN model is only 0.71, the sensitivities of these advanced CNN models are all higher than 0.73, and the DenseNet121 model gets the highest sensitivity of 0.81. The result shows that the sensitivities of the outlined advanced CNN models are better than the basic CNN model.

F1-Score is a statics metric to evaluate the performance of binary classification. The formula of the F1-score tells that its best score is 1 but its worst score is 0. For predicting stage III, the score of basic CNN model is only 0.69, the F1-scores of these advanced CNN models are all higher than 0.72. For predicting stage IVa, the F1-score of basic CNN model is only 0.72, the recalls of these advanced CNN models are all higher than 0.75.

Accuracy is a critical metric corresponding to the proportion of instances that have been correctly classified. Its best score is 1 and its worst score is 0. For predicting stage III, the accuracy of basic CNN model is only 0.69, the accuracies of these advanced CNN models are all higher than 0.74. For predicting stage IVa, the accuracy of basic CNN model is only 0.71, the accuracies of these advanced CNN models are all higher than 0.73.

**Fig. 5:**
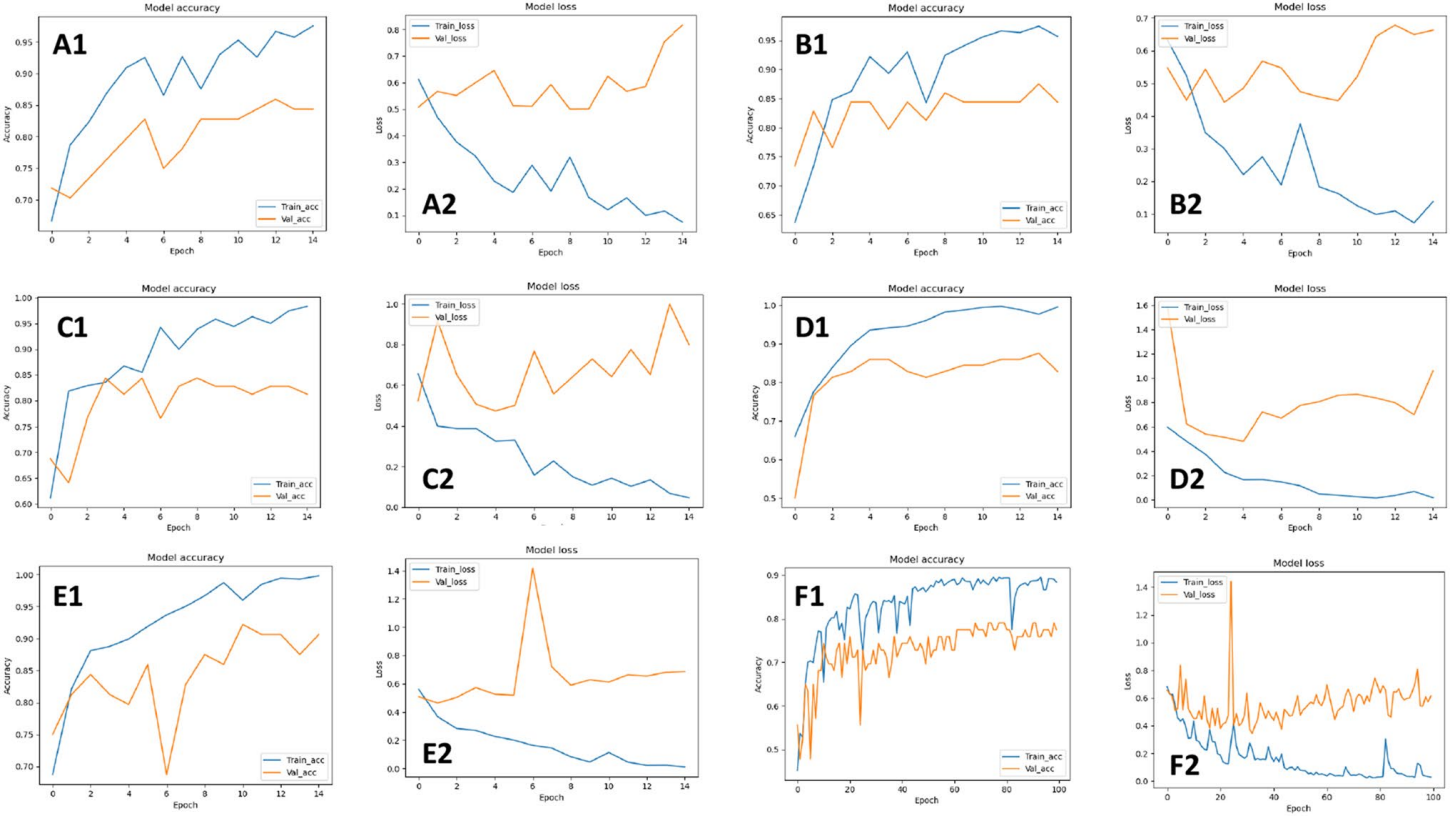
Accuracy and loss curves of the five advanced CNN models. **A1**, **A2**  Curves of VGG16; **B1**, **B2**  curves of InceptionV3; **C1**, **C2**  curves of ResNet50; **D1**, **D2**  curves of InceptionResNetV2; **E1**, **E2**  curves of DenseNet; **F1**, **F2**  curves of basic CNN

The curves of accuracy and loss for training and validation of the five advanced CNN models are shown in Fig. 5. According to these accuracy curves, it is observed that both training and validation accuracies increase as training proceeds, and the training accuracy is slightly higher than the validation accuracy. All accuracies are over 0.7 after 2 epochs, indicating that transfer learning using pretrained models are quite effective for the classifying MR images. After a few epochs, most training accuracies are over 0.8. Moreover, the training losses decrease as the training proceeds. Even though the validation losses do not decrease as expected, the values of the losses are still quite small. However, observing the curves for the basic CNN model, the accuracies for training and validation increase much slow than advanced CNN models, since the accuracies achieve the highest scores after 60 epoches. In addition, the losses for training and validation decrease much slow than advanced CNN models.

According to the experiment results and curves of accuracy and loss, transfer learning is a technique to improve effectiveness and efficiency for NPC stage prediction. Most metrics scores of the advanced CNN models using pretrained models are better than the basic CNN model. The advanced CNN models achieve high accuracies much faster than the basic CNN model, since the models only require less than 10 epoches. These models with pretrained weights are able to capture the low-level features in MR images of LA-NPC, and the fine-tuning is effective to optimize models for binary classification.

#### Effect of Ensemble Model

We give the results of 5 single models and the ensemble model, then tell the difference between these two models. All the testing results of advanced CNN models and our ensemble model are listed in Table 4.

For precision, the ensemble model gets the highest precision of 0.84 to predict stage III of LA-NPC, while the model also gets the highest precision of 0.84 for predicting stage IVa. The result show that the ensemble model achieves the highest precision. Considering sensitivity, the ensemble model gets the highest sensitivity of 0.83 to predict stage III, but the DenseNet121 model gets the highest sensitivity of 0.81 to predict stage IVa and the ensemble model achieves the second highest sensitivity. For F1-score, the ensemble model gets the highest score of 0.81 to predict stage III, and the model gets the highest score of 0.82 to predict stage IVa. For accuracy, the ensemble model gets the highest accuracy of 0.81 to predict stage III, the model gets the highest accuracy of 0.81 to predict stage IVa.

According to the experimental results, the proposed ensemble method achieves the highest accuracy of 0.81. The classification accuracies of advanced CNN models are higher than basic CNN model, while the accuracies of the advanced CNN models are similar to each other. The ensemble model gets the highest accuracies. In addition, the classification precisions for samples of stage IVa are higher than stage III. In general, the ensemble method achieves better scores of evaluation metrics than basic CNN model and most advanced CNN methods.

**Fig. 6:**
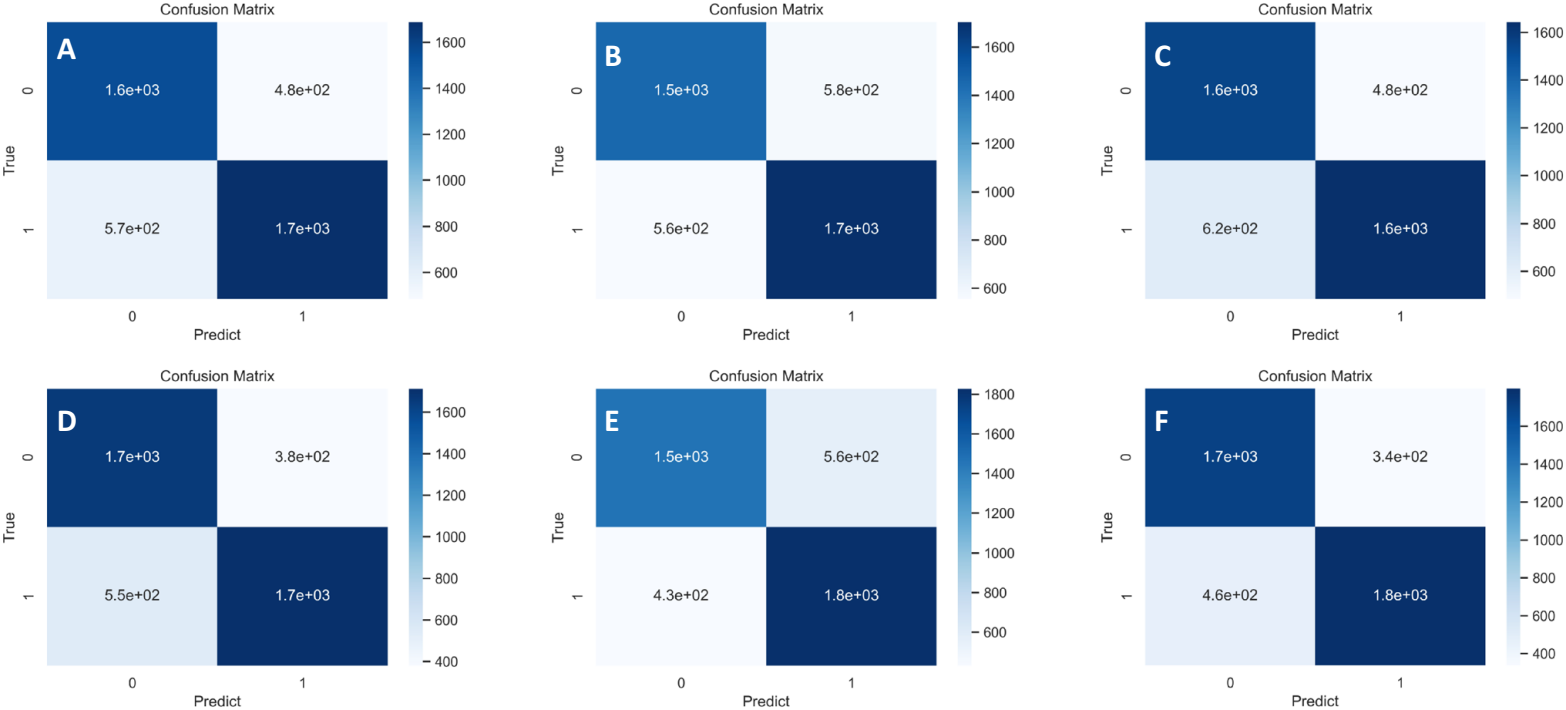
Confusion matrices of the five advanced CNN models and the ensemble model. **A** Confusion matrix of VGG16. **B** Confusion matrix of InceptionV3. **C** Confusion matrix of ResNet. **D** Confusion matrix of InceptionResNetV2. **E** Confusion matrix of DenseNet. **F** Confusion matrix of the ensemble model

The confusion matrices of 5 advanced CNN models and our ensemble model are given in Fig. 6. It is used to tell the performance of classifying LA-NPC as stage III (0/False) and stage IVa (1/True). Each confusion matrix is composed of 4 parts. The TP represents the correctly classified stage III samples, FP represents the stage IVa samples that are misclassified as stage III, TN represents correctly classified stage IVa samples, FN represents stage III samples that are misclassified as stage IVa. For a classification model, the more the TP and TN samples are, the better the performance of the model is. In addition, FP samples are expected to be as few as possible, since stage IVa is the more serious stage which is not expected to be misclassified as stage III for patients. We can see from the confusion matrices that our model gets the most TP and TN samples, while the FP samples are less than most of the other models.

According to the experimental results, ensemble learning is another effective technique in this work to boost the classification performance for stage prediction. Although all the accuracies of advanced CNN models are less than 0.79, the ensemble method using soft voting achieve a higher accuracy of 0.81. Using the same data set, even though these models are all based on convolutional neural network, they are build with different architectures and be able to extract different features from images. The ensemble method benefits from these models and achieves better performance than using single model.

#### Comparisons

Finally, we compare the proposed method with popular ones including TNM staging system and Radiomics method, and the comparison results are listed in Table 5. The evaluation results of TNM staging system and Radiomics are given by experienced clinicians from Renmin hospital. The results tell that the accuracies calculated with TNM staging and Radiomics are only 0.71 and 0.73, while we achieve the accuracy of **0.81** for predicting both stage III and stage IVa, indicating that our method outperforms two classical methods for LA-NPC stages prediction.

**Table 5:** Comparison results

Classes	Methods	Accuracy
Stage III	TNM staging	0.71
	Radiomics	0.73
	Ours	**0.81**
Stage IVa	TNM staging	0.69
	Radiomics	0.71
	Ours	**0.81**

Bold values indicate the highest accuracies using 3 methods for stage III and IVa

### Model Interpretability

**Fig. 7:**
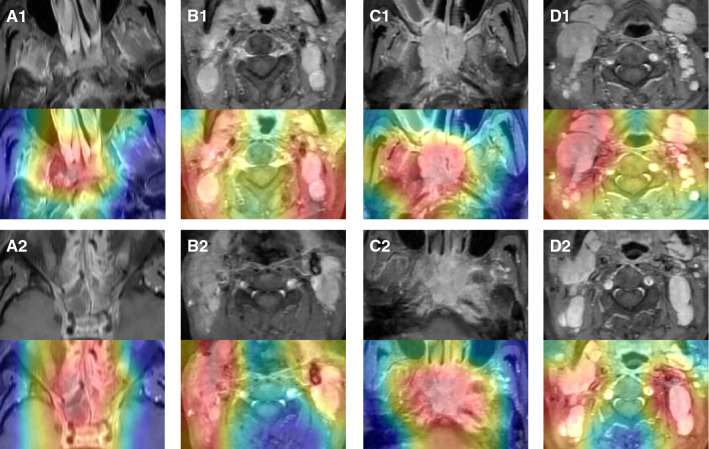
CAM examples for various MR images using InceptionResNetV2. **A1** and **A2** Tumor T2 MRIs of stage III and IVa, **B1** and **B2** lymph node T2 MRI of stage III and IVa, **C1** and **C2** Tumor T1 MRI of stage III and IVa, **D1** and **D2**: lymph node T1 MRI of stage III and IVa

CAM is a technique to produce visual explanations for the decision made using advanced CNN models, making them more interpretable. This technique takes advantage of the gradients of any target object such as tumor and lymph node in this study. The gradients flow into the last CNN layer to produce a heatmap, highlighting the regions of specific classes in the image with different colors. In a heatmap, we may observe four typical colors (red, yellow, green, and blue). The red and yellow regions are strongly related to the prediction result, while the green and blue regions are weekly related. CAM technique can produce heatmaps for all advanced CNN models, and we take the InceptionResNetV2 model as an example to show the heatmaps for various MR images. As shown in Fig. 7, we give heatmaps for tumor of stage III, lymph node of stage III, tumor of stage IVa, and lymph node of stage IVa. According to the observation and analysis by experienced clinicians, it is concluded that the correlations between the tumor/lymph node areas and the red/yellow regions are quite strong, and heatmaps can assist clinicians in LA-NPC diagnosis.

## Conclusion and Future Work

In this paper, we collect a new data set including MR images of LA-NPC patients and introduce a two-stage classification framework base on 2D-CNN models to achieve the LA-NPC stage prediction. Transfer learning is used to improve the classification performance of a single advanced model, an ensemble method is used to further boost the classification performance. We give the detailed steps for data preprocessing, fine-tuning of a single deep learning model, models ensembling, and heatmaps generation. The experiment results show that our method outperforms the TNM staging system and the Radiomics method, as well as the interpretability of our method. In future work, more types of data such as clinical data will be used, and detection methods will be considered to automatically obtain patches of tumors and lymph nodes in full-field MR images.
